# Quality of life perception in type 2 diabetes

**Published:** 2016-11-01

**Authors:** Cristian Petri, Laura Stefani, Vittorio Bini, Nicola Maffulli, Stefania Frau, Gabriele Mascherini, Massimiliano De Angelis, Giorgio Galanti

**Affiliations:** 1 Sports Medicine Center – Clinical and Experimental Medicine Department-School of Sports Medicine University of Florence – Italy; 2 Department of Medicine – University of Perugia – Italy; 3 Department of Musculoskeletal Disorders, Faculty of Medicine and Surgery, University of Salerno, Salerno, Italy; 4 Institute of Health Sciences Education, Centre for Sports and Exercise, Barts and The London School of Medicine and Dentistry, Queen Mary University of London, UK

**Keywords:** diabetes, lifestyle, dietary habits, quality of life, questionnaires

## Abstract

**Purpose:**

Lifestyle analysis is often used for primary and secondary prevention in many chronic metabolic diseases, including diabetes. Questionnaires are simple and common methods for first investigation risk of factors related to the perception of quality of life (QoL). The present study evaluates the feasibility to use questionnaires for first investigation of risk factors, and ascertain whether the results of such questionnaires are associated with the perception of QoL.

**Methods:**

Validated questionnaires from the international ACSM guidelines were used to study a cohort of 142 consecutive type 2 diabetes patients (mean age: 66.1 years ± 10.9).

**Results:**

QoL perception was normal; BMI was compatible with overweight in 79.1% of subjects, and obesity in 20.9%. Cognitive abilities decreased with age and low consumption of dried fruit and legumes. There was evidence of a statistically significant association between BMI and QoL (rho = −0.18; p = 0.03).

**Conclusions:**

Questionnaires are useful to assess lifestyle habits and highlight risks factors. Poor knowledge of patients’ own chronic disease may contribute to a negative impact in diabetes.

## I. INTRODUCTION

Sedentary behavior and inappropriate nutrition habits are ubiquitous in many developed countries[1]. They are often associated with a worse cardio-metabolic risk profile and premature mortality[2]. Lifestyle analysis, including Quality of life (QoL) perception, is a most important aspect in primary and secondary prevention of many non transmissible chronic diseases such as diabetes, hypertension, coronary heart disease, cancer [3,4]. Type 2 diabetes mellitus is strongly related to all-cause and disease-specific mortality. Specifically, when poor lifestyle habits are present [5], the possible association of incorrect nutrition habits to a low physical activity can strongly and negatively impact on QoL. Insufficient physical activity (PA) and a high body mass index (BMI), often associated to incorrect nutrition habits, constitute a global health hazard, given their worldwide prevalence and their combination with increased risk for diseases such as Type 2 diabetes [6]. Abdominal obesity and obesity itself are associated with increased risk of cardiovascular disease [7] in Type 2 diabetes as well as all causes of mortality [8].

Questionnaires are commonly used tools for preliminary investigations in this field to identify sedentarism and poor dietary habits [9,10]. The association between lifestyle habits and anthropometric variables such as, waist circumference (WC) and waist-to hip ratio (WHR) are strongly associated with a low QoL and eventual reduction of many physical and mental abilities, including, for example, capability to attend to the subjects’ own normal daily activities. This could be considered as an early expression of mild a cognitive impairment.

The study aims to detect, in a group of type 2 diabetic patients, the feasibility and the possible role of a dedicated questionnaire to analyze lifestyle, to evaluate the possible association between incorrect lifestyle habits and reduced QOL, as potential mechanism inducing a worsening of the degree of the diabetes itself.

## II. METHODOLOGY

The study protocol was approved by both the Local Ethics Committee of the University of Florence and the Local Ethics Committee of the University of Perugia. All the subjects gave their oral informed consent to participate in the study. Inclusion criteria were the presence of type 2 diabetes for at least 5 years, absence of any other evident co-morbidities such as high-grade hypertension, symptomatic coronary artery disease, and other metabolic diseases.

### Participants

In the period October 2013 to March 2014, 142 consecutive type 2 diabetic patients were recruited. Before enrollment, patients had been regularly followed up at their local diabetic center, and were in stable metabolic condition. Basic anthropometric variables such as BMI, measured as weight/height^2^ expressed in kg/m^2^, abdominal and waist circumferences (expressed in cm) were measured. Patients also answered the SF36 questionnaire to identify their lifestyle habits and quantify their QoL perception The possible presence of cognitive impairment was extrapolated from the SF36 questionnaire.

### Medical Questionnaire

The contents of the questionnaires were extracted from validated models from the international literature (ACSM guidelines) and integrated with Italian questionnaires [11,12].

The questionnaires were composed of three different sections: the first part was composed of questions to evaluate nutrition habits; the second was dedicated to the patients’ own QoL perception and physical activity amount; the third was dedicated to the patients’ knowledge of diabetes.

The three parts of the questionnaire were analyzed, and each score obtained was interpreted separately.

The evaluation of the global QoL perception was based on the analysis of the total amount of the daily free time available, quantity of time dedicated to work or study, time for socialization, time dedicated to physical activity, time for recreational activity, and the perception of the subject’s own cognitive abilities. Four different intervals were considered: > 40 excellent, > 31 < 40 Fair, > 25 < 31 Good, >11 < 25 Unsatisfactory, < 11 Absent. The relevant score was used for each survey [12].

The nutrition habits evaluation was possible directly from the answers expressed in the questionnaire. The data were interpreted following the INRAN (Istituto Nazionale di Ricerca per gli Alimenti e Nutrizione) guidelines.

The evaluation of the patients’ knowledge of diabetes was undertaken using the specific questionnaire where four different topics were investigated and evaluated by the score analysis as follow: glucose (multiple choice questions with maximum value of 11), therapy (with multiple choice questions at maximum value of 6), diabetic foot (up to 10), quality of activities (up to 7). The total score was calculated by the sum of the correct answers. Every correct answer was assigned 1 point. Patients were free not to answer the questionnaire on knowledge of diabetes.

To complete the investigation of diabetes, some hematological parameters were investigated. Glycated Hemoglobin (HbA1c%) was measured, and expressed in % corresponding to the range of IFCC-HbA1c of the International Federation of Clinical Chemistry and Laboratory Medicine (IFCC) standardization (HbA1c Standardization For Clinical Health Care Professionals, NHS Diabetes, Diabetes UK and Association for Clinical Biochemistry 2009), where the range 20–42 mmol/mol is normal. High Density lipoprotein (HDL) cholesterol (mg/dl) and Low Density Lipoprotein cholesterol (mg/dl) level (LDL) were also considered.

Values of HDL cholesterol greater than 40 to 60 mg/dL were considered normal. For LDL cholesterol, a value of 190 mg/dL was considered the cut off point for increased cardiovascular risk. The blood samples for the analysis were obtained early in the morning, at rest, following an overnight fast. All the blood samples were analyzed in batch in the Clinical Pathology Laboratory of the University of Perugia, Italy.

#### Statistical analysis

All data are expressed as mean as SD. The association analyses were performed using the Spearman’s rho test. T student test The Mann-Whitney U test for unpaired data was used to evaluate statistically significant differences. A p value <0.05 was considered statistically significant.

## III. RESULTS

### General data

142 consecutive type 2 diabetes patients mean age: 66.1± 10.9 years; 86 (61.0%) males and 56 women (39.0%). Of them, 13.6% (n=19) were smokers with equal distribution by gender (12.9%) (n=11) males and 14.5% (n=8) females.

### Anthropometrics parameters

All the subjects answered the questionnaire. The mean BMI value, when the population was considered globally, was 29.3 ± 6.0, indication that the population as a whole was overweight, In males, the mean BMI was 28.5 (± 4.2), with 21.2% (n=18) were of normal weight, 44.7% (n=38) overweight, and 34.1% (n=29) were obese. In females, the mean BMI was 29.8 (± 6.6), with 33.3% (n=18) of normal weight, 27.8% (n=15) overweight, and 38.9% (n=21) obese.

### Nutrition habits

**Figure 1:**
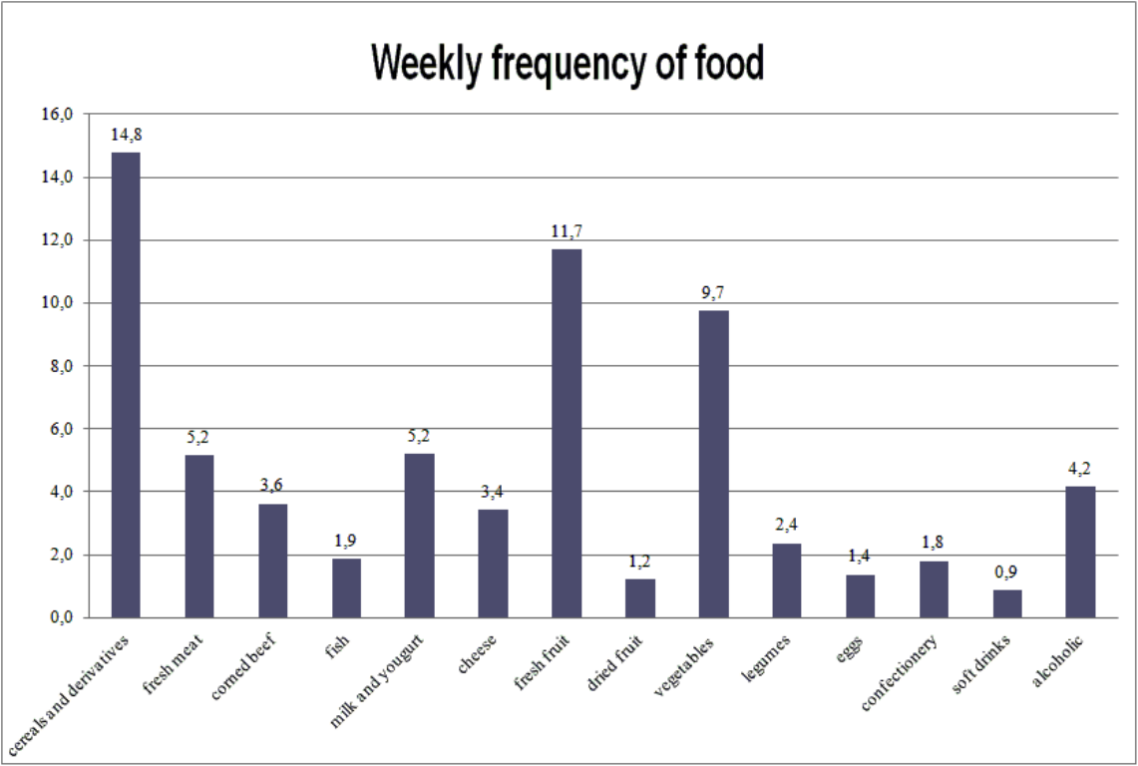
Frequency of consumption of different kind of food.

Four subjects (2.9%) reported food intolerance. The daily consumption of cereals, dairy products, and fresh fruit was high; fish consumption was, on the contrary, low [13] (Figure 1).

### Quality of life perception

**Figure 2:**
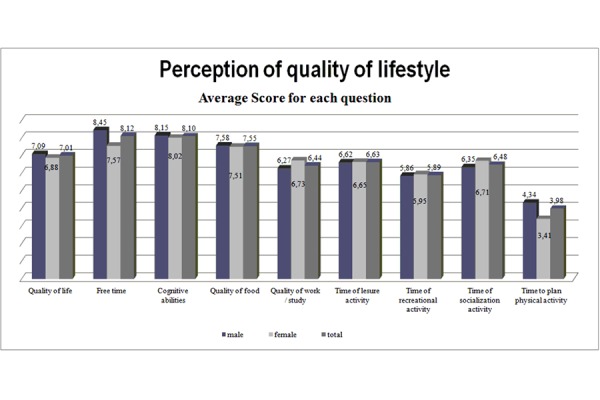
QoL perception analysis and lifestyle habits.

**Figure 3:**
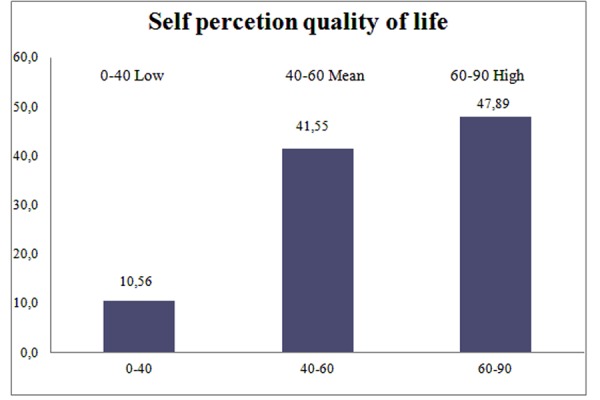
Score of Self perception of quality of life.

Perception of the quality of life showed an average mean score of 56.87 (± 16.90). The mean score was broadly homogenous, with no significant gender differences. The mean score of QOL in women was 52.58 (± 21.8), and 59.6 (± 12.03) in men (Figure 2 and 3).

### Knowledge of diabetes

A total of 75 subjects (52.8%) answered the questionnaire about knowledge of diabetes type 2, with a mean score of 14.36 (± 5.70).

The average scores per each aspect investigated were 5.44 (± 2.64) for the section on blood glucose, 1.92 (± 1.26) for the section on of therapy, 3.97 (± 2.86) for the section on of diabetic foot, and 3.02 (± 1.06) in the section on regarding the QoL.

### Hematological parameters

All the hematological parameters were normal. The mean value of glycosylated hemoglobin was of 6.95±1.28, reflecting a good metabolic balance. Total cholesterol was 177.5 ±44.5 mg/dl, HDL cholesterol was 45±42.67 mg/dl, at the lower limit of normal, and LDL cholesterol was 111 ±29.36 mg/dl.

#### Associations analysis

The associated analysis of nutrition habits with reduced abilities such as early cognitive impairment showed that the higher level of independence in attending their own activities of daily living increases with the consumption of dried fruit (rho= 0.20; p = 0.026). On the contrary, it decreases with the consumption of sweetened drinks (rho= −0.18; p = 0.051). In addition, the decrease of cognitive abilities, such as learning, attention and creative skills, increases with the consumption of cereals and derivatives (rho= −0.22; p = 0.013). The global scores level increases with the consumption of dried fruit (rho=0.20; p = 0.022). Increased BMI is associated with a decrease in quality of lifestyle (rho= −0.18; p = 0.03), a decrease in the quality of nutrition habits (rho=−0.24;p = 0.004), a decrease in the quality and amount of time dedicated in maintaining an active lifestyle (rho= −0.18; p = 0.03), and a decrease in the quality and quantity of time spent in recreational physical activities with friends or family (rho=−0.25; p = 0.004). Moreover, as expected, there was a statistically significant association between progressive increase of age and decrease of some cognitive abilities. This aspect concerned particularly learning, attention, creative abilities (rho=−0.23;p = 0.006), the quality / quantity of work and / or study practiced (rho=−0.21; p = 0.013). It was however associated to a decreased consumption of daily dried fruit (rho=−0.18; p=0.04) and legumes (rho=−0.23; p = 0.008).

Greater self reliance in attending to the activities of daily living was associated to low daily consumption of dried fruit (p = 0.026) and the consumption of carbonated drinks (p = 0.051). On the contrary, the decrease in cognitive abilities, learning, attention, and creative skills was associated to a higher consumption of cereals and derivatives (p = 0.013). In addition, a higher total score in Qol questionnaire was associated to a higher consumption of dried fruit (p = 0.022) (Table 1).

Among the blood parameters investigated, there was an normal Glycated Hb level, HDL cholesterol values at lower level of the normal range, and associated to the High Density Lipoprotein level (p = 0.012) (Table 2).

## IV. DISCUSSION

Correct lifestyle identification is the first step to evaluate the risk of a metabolic chronic disease. Type 2 diabetes is one of the most important conditions accelerating atherosclerosis process, and lifestyle investigations represent the first approach to reduce the cardiovascular risks factors and improve quality of life. Many clinical problems are associated with central obesity [14]. The effect of PA on cardiometabolic risk factors is in fact stronger if concurrent with weight loss. In addition, the perception of Qol plays a relevant role in the possible worsening of a long term conditions such as diabetes [1,2]. The possible incorrect combination of diverse types of food, or, on the contrary, the absence of those nutritional substances with antioxidant power, can, in addition, negatively affect QoL in chronic metabolic disease. Given the importance of daily consumption of fruit, the present data underline the importance to maintain a relatively high intake of fruit given its potential positive effects on the eventual reduction of cognitive abilities. This should be an important take home message also in patients with type 2 diabetes. The importance to investigate this aspect is becoming therefore increasingly widespread [15]. In the 2010 report on the status of non communicable diseases, the World Health Organization estimated that 3.2 million people die each year from failure to engage in correct life style habits. Diabetes is the fourth most important risk factor leading to death in the entire world (6% of all deaths) [16, 17]. Diabetes is one of the most common metabolic chronic diseases, accelerating the process of atherosclerosis, and producing higher mortality [12]. Among the most relevant aspects, proper knowledge of diabetes has a great function to improve its management, but also to improve Qol [18,19]. From the results of the present investigation, it emerges that the progressive decrease of the perception of the patients’ own pathology is often associated to a poor appreciation of their own incorrect lifestyle habits, and therefore a worsening of the metabolic risk’s factors.

This negative circle is particularly relevant in diabetic patients, who have a long life expectancy, and in whom comorbidities can overlap with complications and progression of the original condition.

Despite the apparent global good metabolic control of their diabetes, all the anthropometrics parameters of the patients were compatible with the presence of high cardiovascular risk factors, particularly BMI. Also, nutrition habits were incorrect in terms of the type of food used daily. Moreover, the significant association between the reduced daily abilities, investigated by some questions from SF 36 and as a potential expression of early cognitive dysfunction with the reduction of the antioxidant intake in the population studied, it is particularly relevant in the present investigation. The data highlight the importance of a daily consumption of fruit to maintain good cognitive function.

Particularly regarding the global perception of Qol, despite it being normal in the patients investigated, without any substantial differences by gender, there was a strong relationship with the metabolic risk factors and poor lifestyle habits, including physical inactivity and low level of social-recreational behavior. The patients’ own Qol perception remains in any case a very complex aspect in life style investigation, where many different aspects are strongly related and combined in the long term outcome of the condition.

## V. CONCLUSION

The present study shows that the diabetic patients investigated exhibited a remarkable lack of knowledge of their condition. From the study, the necessity to highlight the role of the patients’ own perception emerges. This would allow more appropriate management of possible complications. The present investigation also confirmed the role of simple questionnaires in evaluating various aspects of Qol perception, including some aspect of the eventual reduction of cognitive function, often not sufficiently considered within the global damage of the metabolic disease. More generally, from the point of view of chronic disease management, the data obtained suggest that the use of questionnaire can allow to plan possible interventions for a positive impact on quality of life and improve health lifestyle in type 2 diabetes patients. Any other specific statement about the possible relationship between cognitive impairments and particular food, like fruit cannot be established from the present investigation.

All these information could be useful to spread the use of questionnaires in Type 2 diabetes patients to better understand the degree and the level of the possible worsening of the condition, and plan individual lifestyle interventions. This is particularly relevant considering that the literature in this field is mainly arising from non-Eurpoean countries, and that the presence of normal glucose plasma levels cannot exclude unhealthy life style habits[20,21]. A more widespread use of questionnaires could be helpful to early identify subjects at risk, and provide more appropriate exercise recommendations to counteract incorrect lifestyle habits. A possible additional use could be to organize structured training, meetings, events, and school camps, making patients more aware and involved in self-care in terms of primary prevention in this field**.**

## Figures and Tables

**Table 1:** Questionnaire regarding knowledge of diabetes.

	Glycemia	Therapy	Diabetic foot	Qol	Total Score
Man	5.7	2.1	3.3	3.0	14.1
Women	5.1	1.7	5.0	3.0	14.8
Total group	5.4	1.9	4.0	3.0	14.4

**Table 2:** Association between of haematological parameters and anthropometrics measurements

			hb%	chol	HDL	LDL
Spearman’s rho	hb%	Correlation Coefficient	1.000	−.218	−.316*	−.076
		Sig. (2-tailed)		.083	.012	.562
		N	74	64	62	60
	chol	Correlation Coefficient	−.218	1.000	.280*	.805**
		Sig. (2-tailed)	.083		.026	.000
		N	64	65	63	61
	HDL	Correlation Coefficient	−.316*	.280*	1.000	.110
		Sig. (2-tailed)	.012	.026		.401
		N	62	63	63	61
	LDL	Correlation Coefficient	−.076	.805**	.110	1.000
		Sig. (2-tailed)	.562	.000	.401	
		N	60	61	61	61

* Correlation is significant at the 0.05 level (2-tailed).

** Correlation is significant at the 0.01 level (2-tailed).
